# The Tumor Border Configuration of Colorectal Cancer as a Histomorphological Prognostic Indicator

**DOI:** 10.3389/fonc.2014.00029

**Published:** 2014-02-18

**Authors:** Viktor H. Koelzer, Alessandro Lugli

**Affiliations:** ^1^Clinical Pathology Division and Translational Research Unit, Institute of Pathology, University of Bern, Bern, Switzerland

**Keywords:** tumor border configuration, invasive margin, tumor growth pattern, infiltrative border, pushing border, colorectal cancer, prognostic factor, tumor–host interaction

## Abstract

Histomorphological features of colorectal cancers (CRC) represent valuable prognostic indicators for clinical decision making. The invasive margin is a central feature for prognostication shaped by the complex processes governing tumor–host interaction. Assessment of the tumor border can be performed on standard paraffin sections and shows promise for integration into the diagnostic routine of gastrointestinal pathology. In aggressive CRC, an extensive dissection of host tissue is seen with loss of a clear tumor–host interface. This pattern, termed “infiltrative tumor border configuration” has been consistently associated with poor survival outcome and early disease recurrence of CRC-patients. In addition, infiltrative tumor growth is frequently associated with presence of adverse clinicopathological features and molecular alterations related to aggressive tumor behavior including BRAFV600 mutation. In contrast, a well-demarcated “pushing” tumor border is seen frequently in CRC-cases with low risk for nodal and distant metastasis. A pushing border is a feature frequently associated with mismatch-repair deficiency and can be used to identify patients for molecular testing. Consequently, assessment of the tumor border configuration as an additional prognostic factor is recommended by the AJCC/UICC to aid the TNM-classification. To promote the assessment of the tumor border configuration in standard practice, consensus criteria on the defining features and method of assessment need to be developed further and tested for inter-observer reproducibility. The development of a standardized quantitative scoring system may lay the basis for verification of the prognostic associations of the tumor growth pattern in multivariate analyses and clinical trials. This article provides a comprehensive review of the diagnostic features, clinicopathological associations, and molecular alterations associated with the tumor border configuration in early stage and advanced CRC.

## Introduction

Prognostication of colorectal cancer (CRC) is based on histopathological staging of the resection specimen according to the AJCC/UICC TNM-classification and guides treatment decisions. The TNM-classification requires reporting of local tumor extent, status of regional nodes, lymphatic and blood vessel invasion, and residual tumor as essential prognostic factors (1). Additional histomorphological indicators are tumor grade, tumor budding, and tumor border configuration (1). Even though the TNM-classification is the gold standard in clinical practice, some patients with lower TNM-stages show a worse prognosis compared to patients with a higher tumor stage. This holds true for selected stage I CRC-patients, some of which paradoxically relapse with nodal metastasis after removal of an early invasive lesion (2). Identification of these patients for segmental resection is of paramount importance for a risk-adapted treatment approach. Further, locally advanced nodal-negative stage II CRC can behave aggressively in the presence of additional histomorphological risk factors (3, 4). In particular, nodal-negative stage II patients with serosal perforation, lymphovascular invasion, perineural infiltration, or an invasive tumor border configuration may have a comparable outcome to stage III patients with nodal positive disease, but do not yet receive neoadjuvant treatment as a standard of care (5–7).

Several recent clinical studies have explored the use of chemotherapeutic treatment in stage II patients with mixed results. A meta-analysis of the available literature by the American Society of Clinical Oncology (ASCO) failed to identify a significant benefit in overall survival of stage II patients receiving adjuvant therapy (8). Consequently, the ASCO does not recommend routine use of chemotherapy in stage II CRC-patients. However, use of adjuvant therapy is considered justified in well-informed patients with additional risk factors such as inadequately sampled nodes, T4 lesions, perforation, or poorly differentiated histology (8). The recent QUASAR trial provides further evidence of an incremental benefit of adjuvant therapy in stage II patients with a significant decrease in disease recurrence and a 3–6% improvement in survival in patients receiving adjuvant chemotherapy (4). From the pathologist’s perspective, the recognition, standardization, and reporting of histomorphological prognostic features are an important basis for identification of patient subgroups with a higher risk of recurrence (9). Clinically, these patients may then benefit from individualized therapeutic approaches and enrollment in clinical trials.

This article provides a comprehensive review on the pathology, biology, and prognostic value of tumor border configuration in CRC. An irregular, “infiltrative” advancing edge is a hallmark of highly aggressive tumors and has been classified as an independent adverse prognostic indicator and may predict propensity for systemic spread of disease in colon and rectal cancer (10–16). In contrast, tumors demonstrating a smooth demarcation with a rounded infiltrative border are classified as having an “expansile” or “pushing” border configuration. As will be discussed in detail, this feature is predictive of limited tumor aggressiveness and is often seen in mismatch-repair (MMR) deficient CRCs (17–19). The addition of tumor border configuration to TNM-staging may help to stratify CRC-patients of a given stage into diagnostic subgroups (7). Importantly, this adverse prognostic feature can be easily detected on standard H&E slides by the histopathologist and is recommended for routine reporting of transmurally invasive CRC as a category IIB prognostic factor (20).

## Surgical Pathology

**Figure 1:**
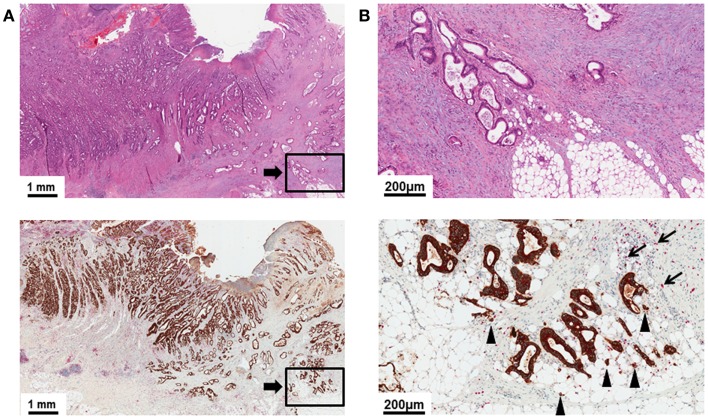
**(A)** Low power image (5×) of a H&E slide and pan-cytokeratin (brown)/CD8 (red) double stain illustrating a transmurally invasive primary CRC with an infiltrative tumor border configuration. Delineation of tumor and host tissue is difficult in the H&E stain. Residual host tissue is present between infiltrating cords and sharp wedges of long-stretched tumor glands. **(B)** High power detail (20×) of **(A)**. Note the diffuse dissection of irregularly shaped tumor glands through the mesenteric adipose tissue. CD8+ lymphocytes at the tumor invasive front are infrequent (arrows). Tumor buds invading the stroma ahead of the invasive front can be identified as a superimposed feature (arrow heads).

**Figure 2:**
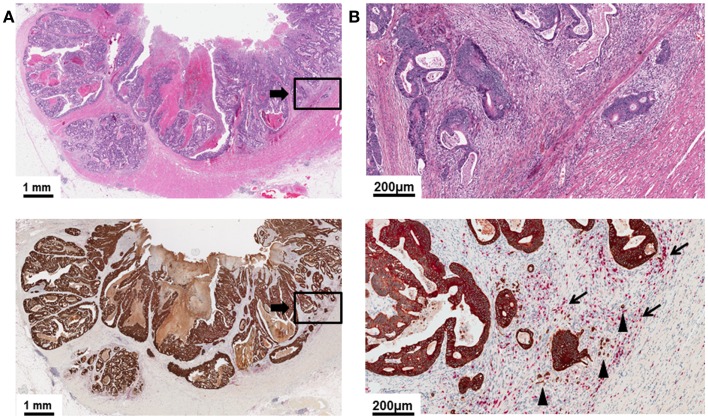
**(A)** Low power image (5×) of a H&E slide and pan-cytokeratin (brown)/CD8 (red) double stain demonstrating a primary CRC with a pushing tumor border configuration of growth. The tumor border is round and well-recognizable at low magnification. Tumor and host tissue can be easily differentiated. Host tissue is displaced by expansile tumor growth. **(B)** High power detail (20×) of **(A)**. CD8+ lymphocytes are commonly observed (arrows). Tumor budding can be recognized as a superimposed feature but is generally infrequent (arrow heads).

The histomorphological variance of the tumor border configuration of CRC was first described by Jass in 1986 as an important histomorphological prognostic indicator in rectal cancer patients (21). Methodologically, tumor border configuration is a feature diagnosed at low magnification and must be clearly differentiated from diagnostic features seen at high power such as tumor budding (10). According to Jass, an infiltrative border configuration should already be suspected when examination of the histopathologic slide with the naked eye does not allow a clear definition of the invasive margin and it seems impossible to resolve host tissue from malignant glands (Figure 1A) (21–23). At low magnification, tumors with an infiltrative growth pattern show dissection of tumor tissue through the anatomic structures of the bowel wall with little or absent desmoplastic stromal response (21–23). The dissecting tumor glands often form irregular clusters or cords of cells, long-stretched glandular structures, or sharp wedges leaving residual host tissue in between, a pattern termed “streaming dissection” (Figure 1B) (10, 11, 23, 24). Presence of perineural invasion on the histologic slide is a further indicator of diffuse infiltration (23). In contrast, a pushing tumor border configuration should be suspected when naked eye examination of the histologic slide allows a clear delineation of the tumor invasive front and host tissue (Figure 2A). Under low magnification, a round “circumscribed” configuration of the infiltrative margin is characteristic of the “pushing” pattern of infiltration (23). Widely dissecting tumor glands in the muscularis propria or mesenteric adipose tissue are absent (Figure 2B).

Infiltrative growth is often observed as a heterogeneously distributed feature in CRC and a majority of cases demonstrate a predominant or at least focally infiltrative tumor border configuration, leading to variability in interpretation. According to Jass, the tumor border configuration is classified as either infiltrating or pushing in a two-tier system (22). This recommendation has been adapted in the CAP reporting standards for CRC (11). However, other authors have advocated the use of a trichotomous classification and to restrict the “infiltrating” pattern to cases with an unequivocal infiltrating growth involving the complete tumor border (25). All of the criteria used to differentiate the pushing from the infiltrative tumor border configuration share a subjective, qualitative nature, and a hierarchy of features have not been designated. This may lead to a very variable classification of cases showing a mixed morphology with either the infiltrating or pushing subset. Further, there is currently no clear consensus on the minimum extension of the infiltrative component necessary to designate a case as having an infiltrative tumor border configuration. While some authors require a predominant infiltrative growth pattern for this classification and describe as few as 17% of CRCs as infiltrative, others have included any case with at least focal infiltrative growth pattern assigning up to 70% of CRC-cases to the infiltrative group (7, 13). Consequently, a suboptimal inter-observer reproducibility has been described for the assessment of tumor border configuration according to the Jass criteria, but can be improved by educating the observers to the defining features (23). Thus, a standardization of the pathologic quantification of the invasive growth pattern is urgently needed to advance its use in daily diagnostic practice.

**Figure 3:**
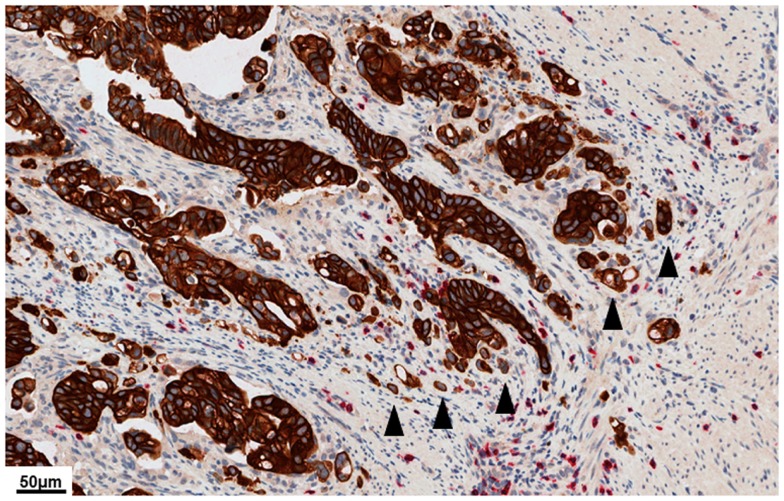
**High power image (40×) of a pan-cytokeratin (brown)/CD8 (red) double stain illustrating dense tumor budding at the tumor invasive front**. Tumor buds (arrow heads) are defined as single cells or small clusters of up to five cells ahead of the tumor invasive front. High-grade tumor budding is a feature of aggressive biological behavior in colorectal cancer. Even though tumor budding is more frequently observed in cases with an infiltrative tumor border configuration, this is an independent feature observed at high power and must not be used to define the quality of the tumor border.

Tumor budding, defined as the presence of single cells or small clusters of up to five cells ahead of the invasive front, is frequently observed as a superimposed pattern in cases with an infiltrative tumor border configuration (Figure 3) (26). However, tumor budding is a separate, independent feature observed at high magnification and must not be used to differentiate infiltrative from pushing tumor growth (9). The presence of tumor budding is an increasingly important histomorphological prognostic factor in CRC (11, 24, 27). Biologically, tumor budding is likely the visible correlate of the process of epithelial mesenchymal transition, during which cancer cells, epithelial by nature, acquire mesenchymal characteristics with capability for migration, stromal lysis, and vascular invasion (28). In early stage CRC, the presence of tumor budding can be indicative of clinical undetectable micrometastases present already at the time of resection of the primary tumor (29). In locally advanced CRC, the presence of tumor budding is an important prognostic indicator for a reduced 5-year survival outcome with elevated risk for disease relapse (27, 30–34). Consequently, tumor budding has been designated a category IIB prognostic factor by the CAP (20).

## Prognostic Impact

### Early stage CRC

Early stage CRC is treated by endoscopic or surgical resection as standard of care. Consequently, the prediction of local recurrence and nodal metastasis by assessment of histopathological features of early invasive CRC is of central importance for patient treatment and follow-up. Several retrospective correlative studies have addressed the prognostic value of the tumor border configuration in early stage CRC (Table 1) and have reported conflicting evidence: in curatively resected submucosally invasive CRC, both Egashira (*n* = 140) and Wang (*n* = 159) failed to identify an association of tumor growth pattern with nodal metastasis (32, 35). In contrast, Keum and colleagues characterize an infiltrating pattern of growth as opposed to a smooth pushing border as a significant independent predictor for disease recurrence in an analysis of 434 patients who underwent curative resection for stage I CRC (36). Further, in an analysis of 111 submucosally invasive pT1 CRC-cases, Akishima-Fukasawa et al. describe the invasive tumor border configuration to be a highly significant predictor of regional lymph node metastasis in univariate but not in multivariate analysis (37). This data is expanded for stage I patients with muscularis propria invasion by Kajiwara and associates who demonstrate an increased frequency of nodal involvement in patients with an infiltrative tumor border configuration in univariate but not in multivariate analysis (38). Taken together, there is a lack of conclusive data on the prognostic value of invasive border configuration in early stage CRC. This may be due to absence of standardized criteria for pathologic assessment. Further, the assessment of the predominant growth pattern in early invasive CRC may be restricted because of limited invasive tumor tissue. As a result, the recommendation for the assessment of tumor border configuration by the AJCC is currently limited to transmurally invasive CRCs (20).

**Table 1:** **Tumor border configuration as a histopathologic risk factor for lymph node metastasis in early stage colorectal cancer**.

Reference	*n*	Infiltrative tumor border configuration (%)	Clinicopathological features analyzed in correlation with pN-stage	Clinicopathological features significantly associated with pN-stage (*p* < 0.05)	Clinicopathological features analyzed with tumor border configuration	Clinicopathological features predicted by infiltrative tumor border configuration (*p* < 0.05)
Egashira (35)	140	Expansile (17.8)	SMd, SMw, differentiation of invasive component, structural atypia of invasive component, border, LInf, L, V, TuB	L1, V1, depth of invasion, cribriform-type structural atypia, low immune infiltration	pN-stage	Not significant (*p* ≥ 0.05)
		Predominant expansile (14.8)	
		Mixed type (42.9)	
		Predominant infiltrating (21.4)	
		Infiltrating (3.5)	
Wang (32)	159	Infiltrating (38.9) Expansile (61.1)	Age, location, CEA-level, Borrmann type, pN-stage, L, V, Pn, G, border, mucinous component, no. of lymph nodes sampled, SMd, LInf, MSI, TuB	G, L1, LInf, TuB	pN-stage survival	Not significantpN-stage (*p* = 0.788)survival (*p* = 0.3328)
						
Kajiwara (38)	244	Infiltrating (27.1) Expansile (72.9)	Age, gender, location, size, TuConf, annularity, G, poorly differentiated component, myxoid cancer stroma, infiltration level in muscularis propria, border, L, V, TuB	Presence of poorly differentiated component, L1, myxoid cancer stroma (multivariate), presence of poorly differentiated component, L1, myxoid cancer stroma, TuB, border (univariate)	pN-stage	pN-stage (*p* = 0.026)
			
Akishima-Fukasawa (37)	111	Infiltrating (46.9) Expansile (53.1)	TuConf, border, disruption of muscularis mucosa, SMd, SMw, G, neutrophil infiltration in cancer cells, fibrotic cancer-type stroma, LInf, Crohn’s-like reaction, microscopic abscess formation, L, V, TuB	Border, disruption of muscularis mucosa, SMd, G, neutrophil infiltration in cancer cells, fibrotic cancer-type stroma, Crohn’s-like reaction, microscopic abscess formation, L1, TuB	pN-stage	pN-stage (*p* = 0.0004)
			
Keum (36)	434	Infiltrating (10.6) Expansile (89.4)	Age, gender, location, size, TuConf, annularity, G, poorly differentiated component, myxoid cancer stroma, infiltration level in muscularis propria, border, L, V, TuB	Infiltrative tumor border, TuB, T2 stage and rectal primary tumor associated with distant tumor recurrence	pN-stage	pN-stage (*p* = 0.017, univariate; *p* = 0.020, multivariate)

*V1, venous invasion; L1, lymphatic invasion; LVI, lymphovascular invasion; G, tumor grade; border, tumor border configuration; TuB, tumor budding, LInf, lymphoid cell infiltrates; R, resection margin status; SMd, depth of submucosal invasion; SMw, width of submucosal invasion; TuConf, macroscopic tumor configuration*.

### Advanced CRC

In transmurally invasive CRC, an infiltrative tumor border configuration has been established as an adverse prognostic factor in several well-designed retrospective cohort studies including CRC-patients of all stages (Table 2). In detail, assessment of the tumor border configuration was found to provide stage-independent prognostic information on the relative risk for disease recurrence and cancer-related death after resection of the primary tumor aiding the TNM- and Dukes-classification (7, 12, 13, 15, 25, 26, 39, 40). The prognostic value of tumor border configuration in locally advanced CRC is supported by several multivariate analyses on independent patient cohorts leading to a recommendation for routine reporting by the CAP (20). Recent data also indicate that the prognostic value of the tumor growth pattern in stage I–III CRC is independent of central molecular features including KRAS, BRAF, and PIK3CA mutations as well as MSI-status and CIMP methylation (25). Assessment of the tumor border configuration should therefore be included in daily diagnostic practice to aid selection of high risk CRC-patients that could derive the most benefit from adjuvant multi-agent therapy.

**Table 2:** **Tumor border configuration as a histopathologic prognostic factor in advanced colon and rectal cancer**.

Reference	Stage	*n*	Infiltrative tumor border configuration (%)	Clinicopathological features predicted by infiltrative tumor border configuration (*p* < 0.05)	End point	Outcome
Jass (21)	I–IV	447	Infiltrating (28.0) Expansile (72.0)	N.A.	Disease specific 5- and 10-year survival rates	Patients with infiltrative growth pattern had a 26% 5-year and a 21% 10-year survival rate, whereas patients with expansile border configuration had a 73% 5-year and a 68% 10-year survival rate (*p* < 0.0001)
Jass (22)	I–IV	379	Not specified	N.A.	Disease specific survival	Infiltrative growth pattern is a significant predictor of shorter disease-specific survival
Halvorsen (12)	I–IV	527	Infiltrating (25.5) Expansile (74.5)	Poor differentiation,	Disease specific 5-year survival rates	Infiltrative growth pattern is an independent predictor of shorter disease-specific survival (HR = 1.64; 95% CI 1.21–2.23)
				Low grade peritumoral inflammatory infiltrate,	
				Low grade peritumoral eosinophil infiltration,	
				Infrequent peritumoral abscess formation, Desmoplasia	
Shepherd (15)	I–IV	251	Infiltrating (16) Expansile (84)	N.A.	Disease specific 5-year survival rates	Infiltrative growth pattern is an independent prognostic variable in patients with extramural spread (*p* < 0.001)
Kubota (13)	I–IV	100	Infiltrating (17) Expansile (83)	N.A.	Cancer-specific and overall survival (mean follow-up 48 months)	Infiltrative growth pattern is an independent predictor of shorter cancer-specific survival (*p* < 0.04; HR = 2.2; 95% CI 1.0–4.6)
Cianchi (41)	I–IV	235	Infiltrating (67.6) Expansile (32.4)	N.A.	Overall 5-year survival rate	Patients with infiltrative growth pattern had a 50.4% 5-year survival rate, whereas patients with expansile border configuration had a 89% 5-year survival rate (*p* < 0.0001)
Ueno (42)	I–IV	638	Infiltrating (17.7) Expansile (73.8)	TuB	Disease specific 5- and 10-year survival rates	5-year survival rates with low grade TuB and infiltrating tumor margin: 49.3%; high-grade TuB: 28.2% (*p* = 0.0389). Five-year survival rates with low grade TuB and expansile tumor margin: 87.0%; high-grade TuB: 54.9% (*p* < 0.001)
Cianchi (43)	IIA	238	Infiltrating (64.4) Expansile (36.6)	N.A.	Disease specific 8-year survival rate	Patients with infiltrative growth pattern had a 72.8% 8-year survival rate, whereas patients with expansile border configuration had a 86.2% 8-year survival rate (*p* < 0.01)
Zlobec (26)	I–IV	1420 (269 with information on local recurrence)	Infiltrating (49.6) Expansile (50.4)	N.A.	Local recurrence	Infiltrative growth pattern is an independent predictor of local recurrence (*p* < 0.001; HR = 3.5; 95% CI 1.8–8.6)
Ueno (39)	II–III	994	Infiltrating (H- or S-spread; 30.48) Expansile (absence of adverse morphology) (69.52)	Vascular invasion Tumor budding Fibrotic stroma	Disease specific 5-year survival rate	Infiltrative growth pattern (H- or S-spread) is an independent predictor of shorter cancer-specific survival (*p* < 0.0096; HR = 1.51; 95% CI 1.11–2.0) Stage II CRC: 5-year survival rate, 81.8% (infiltrating) vs. 92.9% (expansile; *p* = 0.015) Stage III CRC: 5-year survival rate, 57.2% (infiltrating) vs. 78.0% (expansile; *p* < 0.0001)
Zlobec (7)	I–IV	1420	Infiltrating (49.6) Expansile (50.4)	N.A.	Disease specific 5-year survival rate	Infiltrative growth pattern is an independent predictor of shorter cancer-specific survival (HR = 4.75; 95% CI 2.53–8.94). Stage II patients with an infiltrative growth pattern have a decreased disease specific 5-year survival rate (62.7%; 95% CI 48.0–76.2), as compared to patients with an expansile border (82.1%; 95% CI 71.8–90.3)
Zlobec (40)	I–IV	427	Infiltrating (63.9) Expansile (36.1)	Vascular invasion	Disease specific 5-year survival rate	Infiltrative growth pattern is an independent predictor of shorter cancer-specific survival (*p* = 0.004; HR = 1.46; 95% CI 1.1–1.9)
Garcia-Solano (44)	I–IV	162 [81 serrated adenocarcinomas (SAC) and 81 conventional carcinomas (CC)]	SAC: expanding (58.0); infiltrating (42.0) CC: expanding (70.3); infiltrating (29.7)	TuB (SAC, CC) Cytoplasmic pseudofragments (SAC, CC) Regional lymph node metastasis (SAC, CC)	Disease specific 5-year survival rate	SACs with infiltrative growth pattern had a less favorable 5-year survival than expanding SACs (*p* = 0.001) No significant effect in CC (*p* = 0.58)
Huh (45)	I–III	546	Infiltrating (88.1) Expansile (11. 9)	N.A.	5-year disease free survival rate	Decreased 5-year disease free survival rate with infiltrative growth pattern (*p* ≤ 0.05). No significant effect on overall survival
Morikawa (25)	I–IV	1139	Infiltrating (14) Expansile (33) Intermediate (54)	N.A.	Cancer-specific and overall survival (median follow-up 137 months)	Infiltrative growth pattern is an independent predictor of shorter cancer-specific survival (*p* < 0.0001; HR = 1.74; 95% CI 1.22–2.47) and overall survival (*p* < 0.0001; HR = 1.78; 95% CI, 1.33–2.39). Prognostic value of infiltrative growth pattern is limited to stage I–III patients

Several studies have also analyzed the prognostic value of tumor border configuration in locally advanced CRC stratified by stage. The results suggest that additional prognostic information is carried by this feature in patients with stage II, nodal-negative CRC. In a cohort of 238 well-characterized stage IIA CRC-patients, Cianchi et al. provide comprehensive evidence of a significantly elevated risk of cancer-specific death in patients with an infiltrative vs. pushing tumor border configuration: 5-year survival rates are specified as 81.8% with an infiltrating tumor border as opposed to 92.9% in cases demonstrating an expansile growth pattern (*p* < 0.01) (43). Ueno and colleagues confirm these findings in a study of 994 CRC-cases including 434 stage II and 560 stage III patients reporting a 5-year survival rate of 81.8% for the infiltrating group, compared to 92.9% when an expansile border configuration was recorded (*p* = 0.015) (39). This preliminary evidence suggests that the assessment of tumor border configuration may be a promising additional risk-indicator in stage II disease. Assessment of this feature could aid the selection of high risk stage II patients for adjuvant multi-agent therapy and should be investigated further in large randomized multi-center trials to build a reliable evidence base.

The prognostic value of the tumor border configuration in advanced CRC is further supported by correlation with vascular invasion, which is another established feature of aggressive disease. In an analysis of 994 stage II–III patients, Ueno and colleagues describe a strong relation of an infiltrative growth pattern in the muscularis propria to the presence of hemangioinvasion at the invasive front of CRC (39). This data is expanded in a study of 427 stage I–IV CRC-patients by Zlobec and associates who describe a significantly increased frequency of vascular invasion in tumors with an infiltrative growth pattern (40). Further, tumor border configuration was identified as a decisive classifier for the identification of tumors with vascular invasion when combined with expression of Raf-kinase inhibitor protein (RKIP), urokinase plasminogen activator receptor (μPAR), and the proliferative index. As RKIP and μPAR are important biomarkers of tumor motility and invasive capacity, this provides further evidence for an elevated risk of distant metastasis predicted by the tumor growth pattern. Interestingly, an infiltrative tumor border configuration is also strongly predictive of high-grade tumor budding, indicating that the biological processes leading to the formation of tumor buds and an invasive tumor border configuration may be inter-related (39, 44). Patients presenting with both high-grade tumor budding and an infiltrative tumor border configuration have an increased risk of cancer-related death than patients presenting with either alone (39).

Taken together, a solid evidence base for the prognostic value of tumor border configuration exists in advanced CRC and standard reporting of this feature is recommended for diagnostic practice (20). Nevertheless, in order to definitively evaluate the prognostic importance of tumor border configuration in CRC, statistically robust studies with multivariate analysis and standardized criteria for pathologic assessment will be required, particularly for the stage II subset.

## Biological Aspects

The invasive front of CRC embodies the tumor–host interface. Thus, it can be assumed that the histomorphological appearance of this region is the result of the interaction between host and tumor-related factors. Specifically, the dynamics of tumor cell proliferation and expansion is likely governed by specific molecular alterations in cancer cells (46): the individual appearance of the tumor invasive margin will reflect the capacity of tumors to proliferate, migrate, and invade the surrounding stroma. An invasive tumor margin may therefore be the morphological snapshot of directed migration of tumor cells into host tissue after division (47). As tumor cell crowding inhibits growth and proliferation, it can be expected that directed growth also correlates with higher rates of tumor expansion (48). In contrast, a pushing margin may represent the random arrangement of tumor cells after division leading to the formation of static round tumor cell clusters with less propensity or capability for invasion of host tissue. Remarkably, primary CRC with a pushing margin also preferentially cause capsulated liver metastasis while cases with infiltrative tumor growth tend to form poorly demarcated lesions indicating that the configuration of the tumor margin could be a conserved tumor feature independent of the surrounding type or quality of host tissue (49). Biologically, the formation of an infiltrative margin may therefore be tantamount to the tendency for “directed tumor growth” while a pushing margin could be the visual correlate of “non-directed growth” in CRC.

Interestingly, the configuration of the invasive margin correlates with some of the well-characterized molecular alterations in CRC. Specifically, a well-demarcated, expansile tumor border is a feature frequently seen in MMR-deficient CRC-cases, particularly in the setting of the hereditary non-polyposis colorectal cancer syndrome (HNPCC) (17–19). As only about 15% of CRC-cases follow the microsatellite-instable pathway, morphological features are a valuable tool to specifically select cases for molecular testing (10, 18, 50). If a well-defined pushing margin in addition to mucinous differentiation, solid growth pattern, presence of intraepithelial lymphocytes, and Crohn’s-like lymphocytic reaction is observed in a case with right-sided location, the threshold for testing of microsatellite instability should be particularity low as these morphological features reach a sensitivity of 78% and specificity of up 93% for MMR-deficiency (18). In contrast, an infiltrating tumor growth pattern is significantly more frequent in tumors with activating BRAF-mutations, while no impact of KRAS-mutations was observed (25). While MMR-deficient CRC generally has a favorable outcome, BRAF is an independent predictor of an aggressive clinical course (51–53). Interestingly, early functional data indicates that constitutive activation of BRAF may increase the migratory and invasive capacity of human colon cancer cells (54). This could contribute to the significantly more frequent invasive growth pattern observed in CRC-cases with activating BRAF-mutations.

Host-related factors may also influence the appearance of the tumor border in CRC. In a well-designed retrospective study of 527 patients, Halvorsen et al. describe a marked absence of peritumoral inflammation in patients with an infiltrative tumor border configuration (12). In contrast, CRC-cases with a pushing border have a well-characterized association with dense peritumoral inflammatory infiltrates (17–19). Interestingly, it is now well-known that the quantity and quality of peritumoral inflammatory infiltrates is a significant independent predictor of survival outcome and related to the efficiency of the anti-tumoral host response, which may be a possible confounding factor of the prognostic benefit of a pushing tumor border configuration (55–57). Additionally, both features are characteristic of MMR-deficiency, which may be a further hidden variable in early reports (17–19). Both points have been addressed in recent studies. In an analysis of MMR-proficient 269 patients, Zlobec and colleagues demonstrate that an infiltrative tumor border configuration maintains association with local recurrence of CRC independently of the quantity of CD8+ tumor-infiltrating lymphocytes (TIL) and nodal status (26). In detail, node-negative, TIL-negative patients with an infiltrative border configuration had a probability of 0.55 for recurrence whereas patients with a pushing border had a significantly reduced probability of disease relapse of 0.26 (26). In a study of 1139 well-characterized CRC-patients, Morikawa and associates recently confirmed the absence of a modifying effect of peritumoral lymphocytic infiltration on the prognostic value of the tumor growth pattern (25).

Taken together, the tumor border is shaped both by tumor- and host-related factors. A strong correlation with specific molecular alterations has been described, providing further evidence that specific molecular alterations may dictate the growth dynamics of CRC. Importantly, specific BRAF-mutations and MMR-deficiency are also predictive of response to targeted therapy with 5-FU and Cetuximab-based therapy (58–60). Apart from the well-described prognostic associations the tumor growth pattern could therefore also represent a surrogate morphological indicator for specific molecular mutations with predictive value.

## Conclusion

Evaluation of the tumor border configuration by the histopathologist identifies distinct subsets of primary CRC (Table 3) and represents an important histomorphological prognostic indicator. It can be assessed on standard H&E slides and is consistently related to survival outcome of patients with transmurally invasive CRC in statistically robust studies and multivariate analysis. However, the configuration of the tumor border is rarely reported in daily diagnostic practice as its significance for prognostication in CRC is not commonly recognized. Further, the subjective nature and lack of standardization of the assessment method may limit frequent reporting of this important feature in clinical practice. The assessment of the tumor border configuration according to the Jass criteria may profit from the development of a standardized quantitative system and needs to be tested further for reproducibility. Methodologically, this may improve the current substantial variation in assessment criteria between different authors and may lay the basis for inclusion of tumor border configuration as a feature for risk assessment in prospective randomized clinical trials.

**Table 3:** **Contrasting features of primary colorectal cancers depending on the tumor border configuration**.

Characteristics	Infiltrative	Pushing
Examination of the histopathologic slide with the naked eye	No clear definition of the invasive margin is possible (23) Difficulty in resolving host tissue from malignant glands (23)	Clearly recognizable invasive margin Easy delineation of host and malignant tissue
		
Low power magnification	Dissection of tumor tissue through anatomic structures of the bowel wall with little or absent desmoplastic stromal response (23)	“Circumscribed” configuration of the infiltrative margin with desmoplastic stromal reaction
	Irregular clusters, cords, or sharp wedges of long-stretched tumor glands (23)	Absence of widely dissecting tumor glands
	Residual host tissue is present between infiltrating glands “Streaming dissection” (23)	Host tissue is displaced “Expansile tumor growth”
		
Additional features	Presence of perineural invasion (23)	Absence of perineural infiltration
	High-grade tumor budding is frequently observed (26)	Low grade tumor budding is frequently observed
	Peritumoral infiltration less pronounced (12)	Often intense peritumoral inflammation
Molecular pathology features	More frequently observed in cases with activating BRAF-mutations (25)	More frequently observed in cases with microsatellite instability (17–19)
Impact on clinical outcome in transmurally invasive CRC	Unfavorable	Favorable

In early invasive disease, recent studies identify promising associations with clinical outcome. However, evaluation in large, randomized multi-center studies will be needed to conclusively address possible associations of tumor growth pattern with outcome in stage I CRC. For stage II CRC, the evidence base is more consistent making the tumor growth pattern a promising marker to identify high risk patients with locally advanced nodal-negative disease for individualized therapy approaches. However, verification in statistically robust studies with a specific focus on stage II patients is clearly needed.

The association of tumor growth pattern with molecular features of CRC represents an interesting vantage point for further study. In particular, factors determining the directed growth of CRC cells into host tissue remain to be determined. Further, the influence of BRAF-mutations and MMR-deficiency on tumor growth dynamics and plasticity may provide valuable insights into the biology of CRC.

## Conflict of Interest Statement

The authors declare that the research was conducted in the absence of any commercial or financial relationships that could be construed as a potential conflict of interest.
